# Association Between Income and Perinatal Mortality in the Netherlands Across Gestational Age

**DOI:** 10.1001/jamanetworkopen.2021.32124

**Published:** 2021-11-02

**Authors:** Joaquim Vidiella-Martin, Jasper V. Been, Eddy Van Doorslaer, Pilar García-Gómez, Tom Van Ourti

**Affiliations:** 1 Erasmus School of Economics, Tinbergen Institute and Erasmus Centre for Health Economics Rotterdam, Rotterdam, the Netherlands; 2 Centre for Health Service Economics and Organisation, Nuffield Department of Primary Care Health Sciences, University of Oxford, Oxford, United Kingdom; 3 Division of Neonatology, Department of Paediatrics, Erasmus MC Sophia Children’s Hospital, University Medical Centre Rotterdam, Rotterdam, the Netherlands; 4 Division of Obstetrics and Fetal Medicine, Department of Obstetrics and Gynaecology, Erasmus MC Sophia Children’s Hospital, University Medical Centre Rotterdam, Rotterdam, the Netherlands; 5 Department of Public Health, Erasmus MC, University Medical Centre Rotterdam, Rotterdam, the Netherlands; 6 Erasmus School of Health Policy and Management, Rotterdam, the Netherlands

## Abstract

**Importance:**

The association between household income and perinatal health outcomes has been understudied. Examining disparities in perinatal mortality within strata of gestational age and before and after adjusting for birth weight centile can reveal how the income gradient is associated with gestational age, birth weight, and perinatal mortality.

**Objectives:**

To investigate the association between household income and perinatal mortality, separately by gestational age strata and time of death, and the potential role of birth weight centile in mediating this association.

**Design, Setting, and Participants:**

This cross-sectional study used individually linked data of all registered births in the Netherlands with household-level income tax records. Singletons born between January 1, 2004, and December 31, 2016, at 24 weeks to 41 weeks 6 days of gestation with complete information on birth outcomes and maternal characteristics were studied. Data analysis was performed from March 1, 2018, to August 30, 2021.

**Exposures:**

Household income rank (adjusted for household size).

**Main Outcomes and Measures:**

Perinatal mortality, stillbirth (at ≥24 weeks of gestation), and early neonatal mortality (at ≤7 days after birth). Disparities were expressed as bottom-to-top ratios of projected mortality among newborns with the poorest 1% of households vs those with the richest 1% of households. Generalized additive models stratified by gestational age categories, adjusted for potential confounding by maternal age at birth, maternal ethnicity, parity, sex, and year of birth, were used. Birth weight centile was included as a potential mediator.

**Results:**

Among 2 036 431 singletons in this study (1 043 999 [51.3%] males; 1 496 579 [73.5%] with mother of Dutch ethnicity), 121 010 (5.9%) were born before 37 weeks of gestation, and 10 453 (5.1 deaths per 1000) died during the perinatal period. Higher household income was positively associated with higher rates of perinatal survival, with an unadjusted bottom-to-top ratio of 2.11 (95% CI, 1.83-2.44). The bottom-to-top ratio decreased to 1.29 (95% CI, 1.21-1.37) after adjustment for potential confounding factors and inclusion of birth weight centile as a possible mediator. The fully adjusted ratios were similar for stillbirths (1.27; 95% CI, 1.20-1.36) and for early neonatal deaths (1.20; 95% CI, 1.09-1.34). Inequalities in perinatal mortality were found for newborns at greater than 26 weeks of gestation but not between 24 and 26 weeks of gestation (fully adjusted bottom-to-top ratio, 0.87; 95% CI, 0.76-1.00).

**Conclusions and Relevance:**

The results of this large nationally representative cross-sectional study suggest that a large part of the increased risk of perinatal mortality among low-income women is mediated via a lower birth weight centile. The absence of disparities at very low gestational ages suggests that income-related risk factors for perinatal mortality are less prominent at very low gestational ages. Further research should aim to understand which factors associated with preterm birth and lower birth weight can reduce inequalities in perinatal mortality.

## Key Points

**Question:**

Is higher household income associated with perinatal survival in a country with universal access to perinatal care and is this association constant across gestational age strata and by live vs stillbirths?

**Findings:**

In this cross-sectional study of 2 036 431 singleton births in the Netherlands (2004-2016), higher household income was generally associated with lower risk of stillbirth and lower rates of early neonatal death. Higher household income was associated with higher rates of perinatal survival at higher but not at lower gestational age.

**Meaning:**

These findings provide support for continuing public health action to reduce perinatal inequalities and for further research efforts to identify the underlying mechanisms of the association between socioeconomic status and health outcomes.

## Teaser

This cross-sectional study of registered births in the Netherlands examines whether higher household income is associated with perinatal survival.

## Introduction

The global stillbirth rate in 2015 was 14.9 per 1000 births.^1^ Similarly, neonatal death affects 18.6 of every 1000 live births, ranging from 2.8 per 1000 in high sociodemographic index (SDI) countries to 27.7 per 1000 in low SDI countries.^1^ Two of every 5 deaths in children younger than 5 years occur in the neonatal period.^1^

The risk of stillbirth and neonatal death is unevenly distributed not only across but also within countries,^1,2^ even in those with high mean income.^3^ The contribution of socioeconomic status to these disparities has received a lot of attention.^4,5,6,7,8,9^ However, several key aspects of this association remain unexplored. First, the association between household income and perinatal health outcomes has been understudied. The use of household-level income avoids the risk of aggregation bias typically found in studies of associations based on aggregate proxies of socioeconomic status (eg, mean income of a larger geographic area or neighborhood deprivation scores).^10^ Second, the shape of the association between household income and perinatal mortality (often referred to as the income gradient^11^) is unknown and may be nonlinear. Recent studies^12,13^ on the association between income and life expectancy have typically found it to weaken at higher income levels. We are unaware of any such studies in the context of perinatal health. Third, although income-related disparities in perinatal mortality are at least in part explained by low-income mothers delivering more premature and smaller infants,^5,14^ the contributions of gestational age and birth weight are largely unknown. Examining disparities in perinatal mortality *within* strata of gestational age and before and after adjusting for birth weight centile can reveal how the income gradient is associated with gestational age and birth weight.

The Netherlands provides an interesting case study because access to health care is universal and free of charge^15^ and access to perinatal care is fully covered by basic mandatory health insurance. European comparisons from 2003 and 2008 reported high perinatal mortality rates in the Netherlands compared with other European countries but with an improving trend since 2010, further confirmed by the latest report in 2015.^16,17,18,19^ However, concerns about within-country disparities have increased in recent years. Previous evidence^20,21,22,23,24,25,26^ has documented decreasing absolute socioeconomic inequalities in perinatal mortality at the neighborhood level, with inequalities remaining constant despite considerable policy efforts and interventions.

In this study, we examined the association between household-level income and perinatal mortality in the Netherlands from 2004 to 2016. We estimated the shape of this association before and after adjusting for potential confounding factors, such as parity, ethnicity, and maternal age at birth, and evaluated the role of birth weight centile as a potential mediator in this association. In follow-up analyses, we conducted stratified analyses to evaluate disparities within gestational age strata.

## Methods

This cross-sectional study used anonymized data from all registered births in the Netherlands and household-level tax records from January 1, 2004, to December 31, 2016. Data analysis was performed from March 1, 2018, to August 30, 2021. Informed consent is not required in the Netherlands when anonymized data are used. Statistics Netherlands approved the scope of the research and ensured that no personal information was disclosed. Approval to conduct the study was obtained from Statistics Netherlands and Perined, a linked database that combines medical registries from 4 professional groups who provide perinatal care (general practitioner, midwives, gynecologists, and neonatologists/pediatricians). The study followed the Strengthening the Reporting of Observational Studies in Epidemiology (STROBE) reporting guideline.

### Data Sources

Data on all singleton births from 2004 to 2016 of neonates (including stillbirths) born at 24 weeks to 41 weeks 6 days of gestation were obtained from a linked data set of national administrative data accessed remotely via Statistics Netherlands. The Dutch perinatal register (Perined) contains information on maternal characteristics, pregnancy, delivery, and neonatal outcomes for more than 97% of all births in the Netherlands.^27^ Annual household income at the individual level was obtained from the Dutch taxation registry.^28^ A detailed description of the linkage procedures among different administrative data sets can be found in the Statistics Netherlands microdata portal.^29^

### Exposures

Household income rank was used as the main exposure variable. Annual disposable income of the mother’s household, adjusted by household size, was used to rank households in centiles from 1 (lowest) to 100 (highest) relative to the distribution of households with a childbirth for each year separately. Unlike absolute income levels, yearly income ranks are uniformly distributed and more easily compared over time. Household size was accounted for using the modified equivalence scale of the Organization for Economic Cooperation and Development.^30^ The disposable income of a household consists of the gross income, including income transfers, but net of contributions and taxes, such as social security contributions, health insurance, and taxes on income.^28^ We used the income of the year before delivery to prevent the equivalence scale being affected by infant survival.

### Outcomes

The primary outcome was perinatal mortality, defined as intrauterine death occurring after 24 completed weeks of gestational age (stillbirth) or death up to 7 days after live birth (early neonatal death). In separate analyses, perinatal mortality was split into stillbirths (expressed per 1000 total births) and early neonatal mortality (expressed per 1000 live births).

### Potential Confounders

Maternal characteristics included as potential confounders in the analyses were maternal age at delivery (in years), parity (primiparous vs multiparous), and maternal ethnicity. Maternal ethnicity was classified by Perined into 1 of the following 6 categories: Dutch, Moroccan, Turkish, Surinamese, Dutch Antilleans and Arubans, other non-Western countries, and other Western countries. Two other potential confounders were added to our model: year of birth (to account for secular trends in perinatal mortality) and sex of the newborn. The selection of potential confounders was based on data availability and recent other work with Perined data.^26^ Birth weight centile, adjusted for gestational age and sex, was included as a potential mediator of the association between maternal household income rank and perinatal mortality, and was assigned according to the Hoftiezer national reference curves, which are representative of the Dutch population (including migrants).^31^ Gestational age was categorized as follows: 24 to 25 weeks 6 days, 26 weeks to 27 weeks 6 days, 28 weeks to 31 weeks 6 days, 32 weeks to 36 weeks 6 days, and 37 weeks to 41 weeks 6 days and was included as a stratifier in the analysis.

### Statistical Analysis

Maternal characteristics and birth outcomes were tabulated by income quintile and gestational age category to examine the distribution of potential confounding factors over subgroups. To examine income disparities in perinatal mortality, a generalized additive model with binomial response and a logistic link^32^ was specified for each of the 3 mortality outcomes (perinatal death, stillbirth, and early neonatal mortality). This semiparametric approach allowed for the partial association between continuous variables (household income rank, maternal age, and birth weight centile) and the outcome variable to take any form (ie, it allows household income rank and any other continuous variable to be positively and negatively associated with the outcome variable at different points of the household income rank distribution and for the strength of this association to vary across the income distribution).

Estimation proceeded in a stepwise manner. First, mortality was modeled as a function of household income rank. Second, maternal age, maternal ethnicity, parity, child’s sex, and year of birth were added as potential confounders. Third, birth weight centile was added to explore its role as a mediator of the association between household income rank and perinatal mortality.

The mean mortality per household income rank and projected values by income rank were plotted to visually assess the fit of our models. For the models in step 2 and 3, projected values by income rank were obtained after standardizing by potential confounders and/or mediators. Next, we estimated relative inequalities by calculating bottom-to-top ratios of the projected outcomes. These are ratios of projected mortality for individuals with the lowest income rank (1) over mortality for individuals with the highest income rank (100). Values larger than 1 imply that the lowest-income households face higher mortality rates than the highest-income ones, whereas values smaller than 1 indicate that the highest-income households face higher mortality rates than the lowest-income ones. Statistical significance was set at *P* < .05 and was evaluated using a 2-sided test, and 95% CIs were estimated using a parametric bootstrap approach. We sampled 1000 draws of the posterior distribution of the estimates, and the 95% CIs represent the range of values between the ordinal 25th and 975th draws.

To evaluate whether the association between household income rank and perinatal mortality varied by gestational age, stratified analyses were conducted using the gestational age categories defined in the previous section. To facilitate comparison with previous studies in a similar setting,^26^ a sensitivity analysis was performed estimating the same bottom-to-top ratios for the lowest and highest income quintile. To assess how the results of our preferred modeling strategy compare to more conventional estimation methods, a sensitivity analysis was performed using a logistic regression. Furthermore, to evaluate whether region-level measures of mean income attenuate estimated disparities relative to household-level measures of income, a sensitivity analysis was performed with postal code–level mean household income rank as the exposure. To account for potential differences in fetal growth by ethnicity and potential bias in the birth weight centile reference curves, an additional analysis was performed that included only women classified by Perined as ethnically Dutch. Finally, the role of gestational age as a mediator was explored for neonatal mortality as a secondary analysis. Because gestational age is determined at the time of a stillbirth and can therefore simultaneously affect 2 factors in the model, we did not include them in this analysis. All analyses were performed using R, version 3.3.3^33^ and estimated using the *mgcv* package.^34^

## Results

Between 2004 and 2016, a total of 2 228 348 births were registered in Perined. After exclusion of neonates born outside the 24 weeks to 41 weeks 6 days of gestation range, multiple births, missing birth outcomes, and missing tax information, individual-level data on 2 036 431 births (1 043 999 [51.3%] male; 1 496 579 [73.5%] with mother of Dutch ethnicity) were available for analysis (eFigure 1 in the Supplement). No data were imputed for the analyses. A total of 121 010 (5.9%) were born preterm (before 37 weeks of gestation), and 10 453 (5.1 per 1000) died during the perinatal period (Table 1).

**Table 1.** Population Characteristics of the Singleton Pregnancies by Household Income Quintile^a^

Characteristic	Quintile 1 (n = 409 686)	Quintile 2 (n = 409 581)	Quintile 3 (n = 408 172)	Quintile 4 (n = 405 804)	Quintile 5 (n = 403 188)	All (N = 2 036 431)
**Maternal characteristics**						
Maternal age at birth, mean (SD), y	29.7 (5.7)	30.5 (4.9)	30.8 (4.6)	31.4 (4.3)	32.9 (4)	31.1 (4.9)
Maternal age group, y						
<24	61 661 (15.1)	31 217 (7.6)	20 101 (4.9)	11 497 (2.8)	5424 (1.3)	129 900 (6.4)
24-34	259 468 (63.3)	291 481 (71.2)	300 629 (73.7)	299 593 (73.8)	260 334 (64.6)	1 411 505 (69.3)
35-39	68 960 (16.8)	72 182 (17.6)	75 032 (18.4)	81 667 (20.1)	116 725 (29.0)	414 566 (20.4)
>39	19 597 (4.8)	14 701 (3.6)	12 410 (3.0)	13 047 (3.2)	20 705 (5.1)	80 460 (4.0)
Primiparous	123 959 (30.3)	124 654 (30.4)	179 313 (43.9)	234 726 (57.8)	250 921 (62.2)	913 573 (44.9)
Ethnicity						
Dutch	200 585 (49.0)	296 243 (72.3)	328 328 (80.4)	338 196 (83.3)	333 227 (82.6)	1 496 579 (73.5)
Moroccan	43 391 (10.6)	21 067 (5.1)	11 641 (2.9)	7571 (1.9)	4015 (1.0)	87 685 (4.3)
Turkish	28 964 (7.1)	19 175 (4.7)	11 332 (2.8)	6587 (1.6)	3756 (0.9)	69 814 (3.4)
Surinamese	19 390 (4.7)	12 134 (3.0)	8969 (2.2)	7500 (1.8)	5935 (1.5)	53 928 (2.6)
Antillean	12 084 (2.9)	4555 (1.1)	2931 (0.7)	2479 (0.6)	2395 (0.6)	24 444 (1.2)
Other non-Western ethnicities	62 075 (15.2)	21 133 (5.2)	12 644 (3.1)	10 657 (2.6)	12 312 (3.1)	118 821 (5.8)
Western ethnicity	43 197 (10.5)	35 274 (8.6)	32 327 (7.9)	32 814 (8.1)	41 548 (10.3)	185 160 (9.1)
**Pregnancy characteristics**						
Male sex	210 358 (51.3)	209 819 (51.2)	209 285 (51.3)	207 986 (51.3)	206 551 (51.2)	1 043 999 (51.3)
Female sex	199 328 (48.7)	199 762 (48.8)	198 887 (48.7)	197 818 (48.7)	196 637 (48.8)	992 432 (48.7)
Gestational age, mean (SD), wk	39.4 (1.9)	39.5 (1.8)	39.5 (1.8)	39.5 (1.8)	39.6 (1.8)	39.5 (1.8)
Gestational age group						
24 wk to 25 wk 6 d	702 (0.2)	513 (0.1)	487 (0.1)	429 (0.1)	407 (0.1)	2538 (0.1)
26 wk to 27 wk 6 d	951 (0.2)	710 (0.2)	639 (0.2)	656 (0.2)	568 (0.1)	3524 (0.2)
28 wk to 31 wk 6 d	2806 (0.7)	2291 (0.6)	2328 (0.6)	2361 (0.6)	2244 (0.6)	12 030 (0.6)
32 wk to 36 wk 6 d	21 684 (5.3)	19 472 (4.8)	20 193 (4.9)	21 350 (5.3)	20 219 (5.0)	102 918 (5.1)
37 wk to 41 wk 6 d	383 543 (93.6)	386 595 (94.4)	384 525 (94.2)	381 008 (93.9)	379 750 (94.2)	1 915 421 (94.1)
Birth weight centile, mean (SD)	47.3 (30.0)	51.5 (29.9)	51.9 (29.6)	51 (29.3)	50.8 (29.0)	50.5 (29.6)
Birth weight centile group						
<3	15 421 (3.8)	11 711 (2.9)	10 707 (2.6)	10 308 (2.5)	9319 (2.3)	57 466 (2.8)
<10	52 812 (12.9)	41 521 (10.1)	38 627 (9.5)	38 498 (9.5)	36 952 (9.2)	208 410 (10.2)
>90	40 407 (9.9)	49 573 (12.1)	48 625 (11.9)	43 928 (10.8)	41 354 (10.3)	223 887 (11)
Mortality, per 1000						
Perinatal mortality	2826 (6.9)	2167 (5.3)	1984 (4.9)	1873 (4.6)	1603 (4.0)	10453 (5.1)
Stillbirth	2069 (5.1)	1557 (3.8)	1415 (3.5)	1320 (3.3)	1104 (2.7)	7465 (3.7)
Neonatal death	757 (1.9)	610 (1.5)	569 (1.4)	553 (1.4)	499 (1.2)	2988 (1.5)

^a^ Data are presented as number (percentage) unless otherwise indicated. Quintile 1 represents the lowest-income quintile, and quintile 5 denotes the highest income one.

Characteristics of the population by income quintile are given in Table 1. Maternal age at birth and the percentage of primiparous women increased with income quintile, whereas the share of mothers of non-Western ethnicity decreased from 40.6% in the lowest-income quintile to 7.0% in highest-income quintile. Birth weight centile was comparable among the top 4 quintiles and lowest for the bottom quintile. Mean perinatal mortality was 5.1 per 1000 and decreased with increasing income rank—from 6.9 per 1000 in the lowest quintile to 4.0 per 1000 in the highest. Stillbirths accounted for 71.4% of perinatal deaths. The distribution of gestational age and birth weight centile by income quintile is provided in eFigure 2 and eFigure 3 in the Supplement. Low gestational ages and low birth weight centile were more common among lower-income quintiles.

Summary statistics by gestational age category are reported in Table 2. Mothers of non-Western ethnicity accounted for 28.7% of the most extremely preterm births (between 24 weeks and 25 weeks 6 days of gestation) compared with 17.3% of non-Western mothers in the entire study population. The incidence of perinatal mortality ranged from 641.8 per 1000 for births taking place from 24 weeks to 25 weeks 6 days of gestation to 2.0 per 1000 for those born between 37 weeks and 41 weeks 6 days of gestation. Similar patterns were found for stillbirths (421.2 per 1000 for those with gestational age between 24 weeks and 25 weeks 6 days and 1.4 per 1000 for term births) and neonatal mortality (381.2 per 1000 for those with gestational age between 24 weeks and 25 weeks 6 days and 0.6 per 1000 for term births).

**Table 2.** Population Characteristics of the Singleton Pregnancies by Gestational Age Category^a^

Characteristic	Gestational age of 24 wk to 25 wk 6 d (n = 2538)	Gestational age of 26 wk to 27 wk 6 d (n = 3524)	Gestational age of 28 wk to 31 wk 6 d (n = 12 030)	Gestational age of 32 wk to 36 wk 6 d (n = 102 918)	Gestational age of 37 wk to 41 wk 6 d (n = 1 915 421)	All (N = 2 036 431)
**Maternal characteristics**						
Maternal age at birth, mean (SD), y	30.6 (5.6)	30.6 (5.6)	30.8 (5.3)	30.8 (5.1)	31.1 (4.8)	31.1 (4.9)
Maternal age group, y						
<24	269 (10.6)	363 (10.3)	1090 (9.1)	7961 (7.7)	120 217 (6.3)	129 900 (6.4)
24-34	1646 (64.9)	2257 (64.0)	7993 (66.4)	70 546 (68.5)	1 329 063 (69.4)	1 411 505 (69.3)
35-39	476 (18.8)	703 (19.9)	2318 (19.3)	19 796 (19.2)	391 273 (20.4)	414 566 (20.4)
>39	147 (5.8)	201 (5.7)	629 (5.2)	4615 (4.5)	74 868 (3.9)	80 460 (4.0)
Primiparous	1416 (55.8)	2092 (59.4)	7306 (60.7)	59 484 (57.8)	843 275 (44)	913 573 (44.9)
Ethnicity						
Dutch	1589 (62.6)	2290 (65)	8445 (70.2)	75 577 (73.4)	1 408 678 (73.5)	1 496 579 (73.5)
Moroccan	136 (5.4)	199 (5.6)	485 (4.0)	3635 (3.5)	83 230 (4.3)	87 685 (4.3)
Turkish	106 (4.2)	161 (4.6)	431 (3.6)	3300 (3.2)	65 816 (3.4)	69 814 (3.4)
Surinamese	159 (6.3)	190 (5.4)	583 (4.8)	4032 (3.9)	48 964 (2.6)	53 928 (2.6)
Antillean	99 (3.9)	109 (3.1)	255 (2.1)	1617 (1.6)	22 364 (1.2)	24 444 (1.2)
Other non-Western ethnicities	229 (9.0)	290 (8.2)	783 (6.5)	5749 (5.6)	111 770 (5.8)	118 821 (5.8)
Western ethnicities	220 (8.7)	285 (8.1)	1048 (8.7)	9008 (8.8)	174 599 (9.1)	185 160 (9.1)
Income rank (1-100), mean (SD)^b^	45 (29.5)	45.7 (29.4)	48.6 (29.3)	50.2 (29.1)	50.4 (28.8)	50.3 (28.8)
Income rank quintile^c^						
1	702 (27.7)	951 (27.0)	2806 (23.3)	21 684 (21.1)	383 543 (20)	409 686 (20.1)
2	513 (20.2)	710 (20.1)	2291 (19.0)	19 472 (18.9)	386 595 (20.2)	409 581 (20.1)
3	487 (19.2)	639 (18.1)	2328 (19.4)	20 193 (19.6)	384 525 (20.1)	408 172 (20.0)
4	429 (16.9)	656 (18.6)	2361 (19.6)	21 350 (20.7)	381 008 (19.9)	405 804 (19.9)
5	407 (16.0)	568 (16.1)	2244 (18.7)	20 219 (19.6)	379 750 (19.8)	403 188 (19.8)
**Pregnancy characteristics**						
Male sex	1346 (53.0)	1896 (53.8)	6751 (56.1)	57 004 (55.4)	977 002 (51.0)	1 043 999 (51.3)
Female sex	1192 (47.0)	1628 (46.2)	5279 (43.9)	45 914 (44.6)	938 419 (49.0)	992 432 (48.7)
Birth weight centile, mean (SD)	35.9 (32.6)	31.2 (32.4)	33.1 (32.3)	45.3 (31.4)	50.9 (29.4)	50.5 (29.6)
Birth weight centile group						
<3	567 (22.3)	1038 (29.5)	2685 (22.3)	7804 (7.6)	45 372 (2.4)	57 466 (2.8)
<10	859 (33.8)	1500 (42.6)	4565 (37.9)	18 409 (17.9)	183 077 (9.6)	208 410 (10.2)
>90	195 (7.7)	221 (6.3)	808 (6.7)	10 276 (10.0)	212 387 (11.1)	223 887 (11)
Mortality						
Perinatal mortality	1629 (641.8)	1042 (295.7)	1608 (133.7)	2300 (22.3)	3874 (2.0)	10453 (5.1)
Stillbirth	1069 (421.2)	779 (221.1)	1235 (102.7)	1692 (16.4)	2690 (1.4)	7465 (3.7)
Neonatal death	560 (381.2)	263 (95.8)	373 (34.6)	608 (6.0)	1184 (0.6)	2988 (1.5)

^a^ Data are presented as number (percentage) unless otherwise indicated.

^b^ Income rank is the annual disposable income of the mother’s household, adjusted by household size, and was used to rank households in centiles from 1 (lowest) to 100 (highest) relative to the distribution of households with a childbirth for each year separately.

^c^ Quintile 1 represents the lowest-income quintile, and quintile 5 denotes the highest income one. The size of the quintiles is not identical because household income rank was calculated before excluding neonates born outside the 24 weeks to 41 weeks 6 days of gestation range, multiple births, and missing birth outcomes (eFigure 1 in the Supplement).

Information on 46 151 births that were unlinked because of missing income data is provided in eTable 1 in the Supplement. Newborns with unlinked data were more likely to die in the perinatal period than the study population (7.9 per 1000 vs 5.1 per 1000), and their mothers were less likely to be of Dutch ethnicity (28.2% vs 73.5%) and more likely to be younger than 24 years (19.7% vs 6.4%).

### Income Disparities in Perinatal Mortality

In the unadjusted model, higher household income rank was associated with lower perinatal mortality at the bottom and top of the household income distribution but not in the middle (Figure, A). After adjustment for potential confounders, the income gradient in perinatal mortality became smaller, but it remained negative across the entire household income distribution. Further adjustment for birth weight centile led to an almost linear (consistently negative) association between perinatal mortality and household income rank. The bottom-to-top ratio was 2.11 (95% CI, 1.83-2.44) in the unadjusted model (Table 3). Adjustment for potential confounding factors led to a ratio of 1.74 (95% CI, 1.58-1.92), which was further attenuated after adjusting for birth weight centile (bottom-to-top ratio, 1.29; 95% CI, 1.21-1.37).

**Figure.**
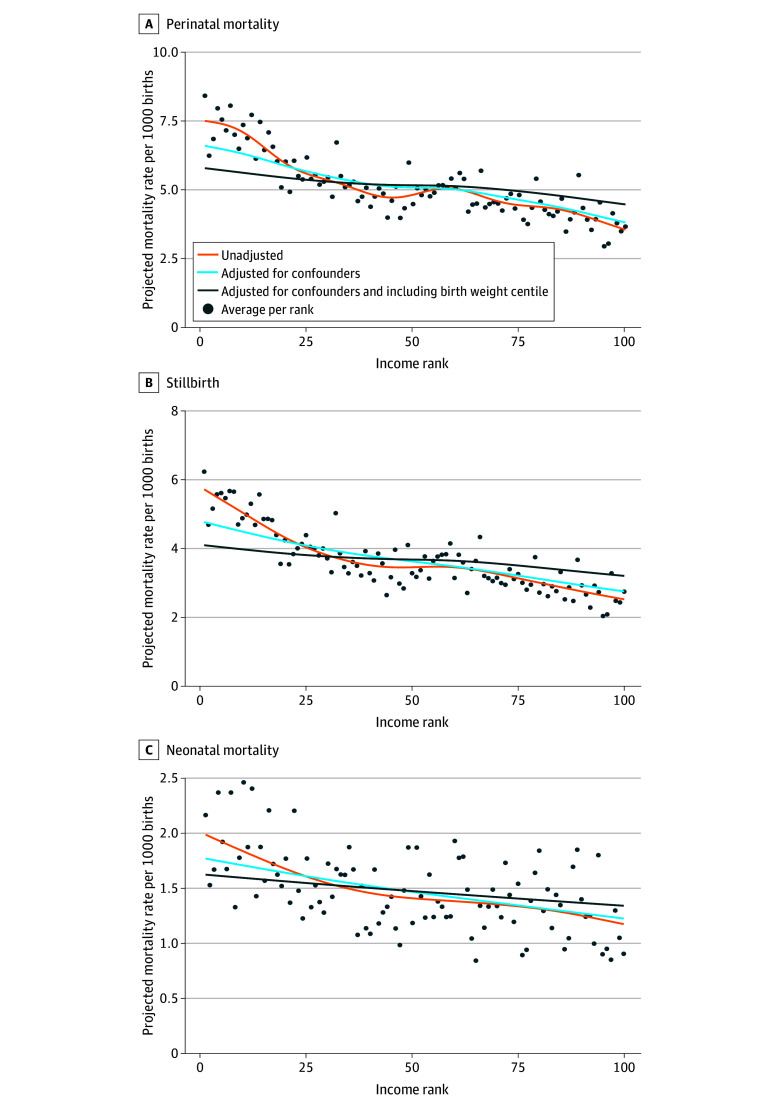
Mean Outcomes per Household Income Rank and Projected Outcomes of the Fitted Models Each dot depicts the mean mortality for each maternal household income rank. Orange lines indicate projected mortality rates before adjustment for potential confounders; light blue lines, projected mortality rates after adjustment for potential confounders; and dark blue lines, projected mortality rates after further including birth weight centile as a potential mediator.

**Table 3.** Estimated Bottom-to-Top Ratios (95% CIs) for Perinatal Mortality, Stillbirths, and Neonatal Mortality for the Pooled Models^a^

Mortality	Unadjusted	Adjusted^b^	Adjusted and BWC
Perinatal mortality	2.11 (1.83-2.44)	1.74 (1.58-1.92)	1.29 (1.21-1.37)
Stillbirth	2.27 (1.98-2.60)	1.75 (1.60-1.92)	1.27 (1.20-1.36)
Neonatal mortality	1.69 (1.44-2.00)	1.45 (1.27-1.66)	1.20 (1.09-1.34)

Abbreviation: BWC, birth weight centile.

^a^ Bottom-to-top ratios divide projected perinatal mortality, stillbirth, and neonatal mortality for the lowest household income rank (1) by the projected one for the highest household income rank (100). Values larger (smaller) than 1 imply that the lowest-income households face higher (lower) mortality rates than the highest-income ones. A value of 1 indicates no difference between outcome variables for the lowest and highest household income ranks.

^b^ Adjusted for maternal age at delivery, maternal ethnicity, parity, sex, and year of birth.

Perinatal mortality was then split into stillbirths (Figure, B) and neonatal mortality (Figure, C). The association between household income rank and each of the 2 outcomes was stronger (ie, more negative) at the bottom of the household income distribution, as shown by the steeper slopes in the figure. This decrease was mostly driven by a reduction of the gradient at the bottom of the household income distribution. Unadjusted bottom-to-top ratios were larger for stillbirths than neonatal mortality, but similar in magnitude adjustment for potential confounders and inclusion of birth weight centile.

### Stratified Analyses by Gestational Age

The results of the stratified analysis are reported in Table 4. In all gestational age categories, except for the one that included births between 24 weeks and 25 weeks 6 days of gestation, the unadjusted bottom-to-top ratios for perinatal mortality were significantly larger than 1, indicating a negative association between household income rank and perinatal mortality (Table 4). After adjustment for potential confounding factors, the estimated ratios were reduced but still significantly larger than 1. After additional adjustment for birth weight centile, the ratios were further reduced but remained significant in all but the two lowest gestational age categories (24 weeks to 25 weeks 6 days, and 26 weeks to 27 weeks 6 days). The ratio was 0.87 (95% CI, 0.76-1.00) for those with the lowest gestational age and 1.22 (95% CI 1.13-1.33) for those with the highest gestational age category.

**Table 4.** Estimated Bottom-to-Top Ratios (95% CIs) for Perinatal Mortality, Stillbirths, and Neonatal Mortality From the Stratified Analysis^a^

Mortality	Unadjusted	Adjusted^b^	Adjusted and BWC
**Perinatal mortality**			
24 wk to 25 wk 6 d	0.91 (0.83-1.01)	0.97 (0.87-1.09)	0.87 (0.76-1.00)
26 wk to 27 wk 6 d	1.36 (1.16-1.61)	1.48 (1.20-1.86)	1.14 (0.98-1.34)
28 wk to 31 wk 6 d	1.68 (1.44-1.99)	1.51 (1.25-1.89)	1.24 (1.08-1.44)
32 wk to 36 wk 6 d	2.37 (2.02-2.75)	1.72 (1.44-2.06)	1.23 (1.10-1.41)
37 wk to 41 wk 6 d	1.55 (1.38-1.75)	1.46 (1.32-1.63)	1.22 (1.13-1.33)
**Stillbirth**			
24 wk to 25 wk 6 d	1.06 (0.92-1.24)	1.17 (0.97-1.41)	0.98 (0.82-1.19)
26 wk to 27 wk 6 d	1.70 (1.36-2.16)	1.88 (1.47-2.54)	1.22 (1.07-1.43)
28 wk to 31 wk 6 d	1.85 (1.54-2.24)	1.66 (1.32-2.12)	1.26 (1.11-1.49)
32 wk to 36 wk 6 d	2.36 (1.97-2.79)	1.76 (1.46-2.18)	1.24 (1.10-1.43)
37 wk to 41 wk 6 d	1.56 (1.35-1.78)	1.44 (1.30-1.64)	1.22 (1.11-1.33)
**Neonatal mortality**			
24 wk to 25 wk 6 d	0.71 (0.58-0.88)	0.78 (0.60-1.00)	0.76 (0.58-0.97)
26 wk to 27 wk 6 d	0.90 (0.62-1.35)	0.69 (0.29-1.41)	0.68 (0.33-1.25)
28 wk to 31 wk 6 d	1.31 (0.97-1.85)	1.03 (0.58-1.80)	1.03 (0.65-1.66)
32 wk to 36 wk 6 d	2.46 (1.87-3.32)	1.57 (0.99-2.46)	1.18 (0.82-1.74)
37 wk to 41 wk 6 d	1.48 (1.22-1.82)	1.49 (1.24-1.89)	1.21 (1.05-1.45)

Abbreviation: BWC, birth weight centile.

^a^ Bottom-to-top ratios divide projected perinatal mortality, stillbirth, and neonatal mortality for the lowest household income rank (1) by the projected one for the highest household income rank (100). Values larger (smaller) than 1 imply that the lowest-income households face higher (lower) mortality rates than the highest-income ones. A value of 1 indicates no difference between outcome variables for the lowest and highest household income ranks.

^b^ Adjusted for maternal age at delivery, maternal ethnicity, parity, sex, and year of birth.

A similar pattern was found for stillbirths (Table 4). The fully adjusted model, including birth weight centile, led to an estimated bottom-to-top ratio of 0.98 (95% CI, 0.82-1.19) in the lowest gestational age category, 1.22 (95% CI, 1.07-1.43) in the 26 weeks to 27 weeks 6 days category, 1.26 (95% CI, 1.11-1.49) in the 28 weeks to 31 weeks 6 days category, 1.24 (95% CI, 1.10-1.43) in the 32 weeks to 36 weeks 6 days category, and 1.22 (95% CI, 1.11-1.33) in the 37 weeks to 41 weeks 6 days category.

Before adjustment, neonatal mortality for births between 24 weeks to 25 weeks 6 days of gestation was positively associated with household income rank, with a bottom-to-top ratio of 0.71 (95% CI, 0.58-0.88) (Table 4). After adjustment for potential confounders and birth weight centile, higher household income rank was still associated with higher neonatal mortality, with a bottom-to-top ratio of 0.76 (95% CI, 0.58-0.97). In the fully adjusted models, no statistically significant association was found for newborns between 26 weeks and 37 weeks of gestation. For term newborns, the bottom-to-top ratio was 1.21 (95% CI, 1.05-1.45).

### Sensitivity Analyses

The lowest- and highest-income quintile ratios are given in eTable 2 and eTable 3 in the Supplement. These results confirmed the findings from the bottom-to-top centile ratios, with increased statistical precision because of the use of a larger share of the sample to calculate them.

The results from the logistic regressions are summarized in eTable 4 and eTable 5 in the Supplement and confirm that associations between household income and perinatal mortality were generally negative and absent at early gestational ages. Because of its parametric assumptions, the logistic-based approach cannot capture the first downward sloping and then upward sloping patterns found in the Figure.

Estimating income-related disparities using postal code–level mean income instead of household income attenuated bottom-to-top centile ratios (eTables 6 and 7 in the Supplement). In the fully adjusted model, including birth weight centile, the estimated bottom-to-top ratios for perinatal mortality, stillbirths, and neonatal mortality were 1.15 (95% CI, 1.11-1.20), 1.14 (95% CI, 1.10-1.19), and 1.15 (95% CI, 1.04-1.29), respectively. Adjustment for birth weight centile led to similar patterns in the estimated disparities when considering Dutch women only (eTables 8 and 9 in the Supplement).

The results of the evaluation of the mediating role of gestational age in neonatal mortality are included in eTable 10 in the Supplement. The estimated bottom-to-top centile ratio was attenuated to 1.07 (95% CI, 1.02-1.14) when including gestational age as a potential mediator compared with 1.20 (95% CI, 1.09-1.34) when including birth weight centile (Table 3).

## Discussion

In this cross-sectional study, we found a negative association between maternal household income rank and perinatal mortality, both before and after adjusting for potential confounding and mediating factors. Lower household income was associated with increased risk of stillbirth and early neonatal death, and this association was only partly mediated by lower birth weight centiles among poorer individuals. Part of the explanation lies in poorer women giving birth at lower gestational ages, but the sign and strength of the within-strata association also varied across gestational age categories: although no significant inequalities in perinatal mortality were present for newborns between 24 weeks and 25 weeks 6 days of gestation, disparities that favored richer individuals were found in all other gestational age groups. These results suggest that in a setting of high medical risk, socioeconomic factors, such as household income, might have less relative influence compared with clinical interventions.

Disparities in stillbirths were consistently and significantly in favor of richer individuals at higher gestational ages, but no significant disparities were present at less than 26 weeks of gestation. This finding contrasts with inequalities in neonatal mortality in favor of poorer individuals at less than 26 weeks of gestation. This more surprising finding may relate to the interaction between critical care and parental income. One possible explanation could be that high-income households may be less amenable to opting for active management of infants born at the edge of viability and for continuation of intensive care in case of severe complications associated with a poor long-term outlook. Previous literature^35,36^ has reported that socioeconomic status may indeed play a role in end-of-life decisions for health care professionals and parents, which is an important topic for future research.

After adjustment for potential confounders and birth weight centile, inequalities in the pooled models remained similar in stillbirths and neonatal mortality. Inequalities were more strongly reduced after adjusting for gestational age, but neonatal mortality inequalities remained in favor of richer individuals. Better understanding of the interaction between perinatal care, income, and risk factors is required to interpret these findings.

Our study builds on previous work^4,5,6,7,8,9,20,21,22,23,24,25,26^ that assessed socioeconomic inequalities in perinatal health. Significant associations between parental socioeconomic status (measured by maternal educational level or income) and perinatal outcomes have been found in Denmark, Canada, and the US, among other countries.^7,37,38^ Our use of household-level data led to markedly larger disparity estimates in perinatal mortality than those reported in a recent Dutch study^26^ using neighborhood-level measures of socioeconomic status. Likewise, reestimating bottom-to-top centile ratios using area- rather than household-level measures of income led to lower disparities in all 3 outcomes (eTables 6 and 7 in the Supplement).

Each additional increase in the household income rank was associated with a larger decrease in mortality at the bottom half of the income distribution than in the top half. This difference between bottom and top half was attenuated but not totally removed after adjusting for potential confounders and mediators, suggesting that income differences at the bottom end are more relevant for explaining perinatal survival.

### Strengths and Limitations

The main strength of our study is the use of a population-wide perinatal register, linked with parental tax records, which includes more than 97% of infants born in the Netherlands and is therefore highly representative. This register enabled us to estimate the magnitude of household income disparities in perinatal mortality without running the risk of aggregation bias typically faced in these studies. Unlinked births because of missing income data were more likely to belong to a vulnerable group (ie, young mother or non-Dutch mother), and these infants more likely die during the perinatal period. This finding suggests that our estimates may provide a lower bound of the inequalities in the overall population. Our semiparametric method allowed us to avoid parametric assumptions commonly imposed when estimating the association between exposure and outcome variables and found that this association can be positive and negative at different points of the income distribution. One limitation of this study is that we had no information on maternal behavior or health care provision during pregnancy, which may mediate the association between household income rank and perinatal mortality^39^ and whose mediating role could differ across gestational age categories. In addition, other household income factors might play a role in this association. For instance, other measures of socioeconomic inequality, such as distance to hospital or neighborhood trajectories, may be associated with perinatal outcomes.^8,40,41^ Moreover, the classification of ethnicity was based on abstractors’ identification of maternal ethnicity. Furthermore, findings from the stratified analysis should be interpreted with caution because of the possibility of collider bias. Finally, the low number of children born in the lowest gestational age strata led to imprecise estimates in the stratified analyses.

## Conclusions

Disparities in perinatal mortality favored richer individuals in 2004 to 2016 in the Netherlands, in particular for stillbirths. Income-related disparities were absent in the lowest gestational age stratum and were largely mediated by birth weight centile. The findings of this study provide support for continuing public health action to reduce perinatal inequalities and for further research efforts to unveil the underlying mechanisms of the association between socioeconomic status and health outcomes.^42^

## Notes

Supplement. **eFigure 1.** Cohort Composition **eFigure 2.** Distribution of Gestational Age and Birth Weight Centile by Household Income Quintile **eFigure 3.** Average Gestational Age and Birth Weight Centile per Household Income Rank and Predicted Outcomes of the Fitted Model **eTable 1.** Descriptive Statistics for Unlinked Observations **eTable 2.** Estimated Bottom-Top Quintile Ratios for Perinatal Mortality, Stillbirths, and Neonatal Mortality for the Pooled Models **eTable 3.** Estimated Bottom-Top Quintile Ratios for Perinatal Mortality, Stillbirths, and Neonatal Mortality From the Stratified Analysis **eTable 4.** Beta Coefficients From Logistic Regressions on Perinatal Mortality, Stillbirth, and Neonatal Mortality for Pooled Models **eTable 5.** Beta Coefficients From Logistic Regressions on Perinatal Mortality, Stillbirth, and Neonatal Mortality for Stratified Models **eTable 6.** Estimated Bottom-Top Ratios for Perinatal Mortality, Stillbirths, and Neonatal Mortality for Pooled Models, Using Area-Level Measures of Income **eTable 7.** Estimated Bottom-Top Ratios for Perinatal Mortality, Stillbirths, and Neonatal Mortality for Stratified Models, Using Area-Level Measures of Income **eTable 8.** Estimated Bottom-Top Ratios for Perinatal Mortality, Stillbirths, and Neonatal Mortality for Pooled Models, Including Only Mothers of Dutch Ethnicity **eTable 9.** Estimated Bottom-Top Ratios for Perinatal Mortality, Stillbirths, and Neonatal Mortality for Stratified Models, Including Only Mothers of Dutch Ethnicity **eTable 10.** Estimated Bottom-Top Ratios for Neonatal Mortality, Including Gestational Age as a Mediator
