# Errors in Data Analysis

**DOI:** 10.1001/jamanetworkopen.2024.42090

**Published:** 2024-10-08

In the Original Investigation titled “Association Between Income and Perinatal Mortality in the Netherlands Across Gestational Age,”^1^ published November 2, 2021, the authors have reported errors requiring correction. The ratio of stillbirths to early neonatal deaths (days 1-7) in the Perined data, the article’s main data source, should have been approximately 2.5 to 3.0 rather than 5.9, which was used in the original calculations. Consequently, deaths occurring between days 2 and 7 were incorrectly coded as live infants. In the Perined data, deaths are categorized as stillbirth, first-day death, or death occurring on days 2 to 7. This coding error specifically affected the perinatal and early neonatal mortality results and descriptive statistics and led to errors in the Abstract, main article, Figure, and Supplement. While the point estimates have changed, the paper’s main conclusions remain valid, particularly regarding the inequalities in perinatal mortality across income levels. The authors have explained the errors and corrections in a Comment posted with the corrected article.^2^

## References

[1] Vidiella-Martin J, Been JV, Van Doorslaer E, García-Gómez P, Van Ourti T. Association between income and perinatal mortality in the Netherlands across gestational age. JAMA Netw Open. 2021;4(11):e2132124. doi:10.1001/jamanetworkopen.2021.32124 PMC8564582 34726746

[2] Vidiella-Martin J, Been JV, Van Doorslaer E, García-Gómez P, Van Ourti T. Association between income and perinatal mortality in the Netherlands across gestational age. *JAMA Network Open*. Accessed October 8, 2024. https://jamanetwork.com/journals/jamanetworkopen/fullarticle/2785667?resultClick=1 10.1001/jamanetworkopen.2021.32124 PMC8564582 34726746

