# Supplementary material for: Association Between Income and Perinatal Mortality in the Netherlands Across Gestational Age
Source: JAMA Netw Open. 2021 Nov 2;4(11):e2132124. doi: 10.1001/jamanetworkopen.2021.32124 (PMC8564582; doi:10.1001/jamanetworkopen.2021.32124)
Supplement: Supplement. — eFigure 1. Cohort Composition eFigure 2. Distribution of Gestational Age and Birth Weight Centile by Household Income Quintile eFigure 3. Average Gestational Age and Birth Weight Centile per Household Income Rank and Predicted Outcomes of the Fitted Model eTable 1. Descriptive Statistics for Unlinked Observations eTable 2. Estimated Bottom-Top Quintile Ratios for Perinatal Mortality, Stillbirths, and Neonatal Mortality for the Pooled Models eTable 3. Estimated Bottom-Top Quintile Ratios for Perinatal Mortality, Stillbirths, and Neonatal Mortality From the Stratified Analysis eTable 4. Beta Coefficients From Logistic Regressions on Perinatal Mortality, Stillbirth, and Neonatal Mortality for Pooled Models eTable 5. Beta Coefficients From Logistic Regressions on Perinatal Mortality, Stillbirth, and Neonatal Mortality for Stratified Models eTable 6. Estimated Bottom-Top Ratios for Perinatal Mortality, Stillbirths, and Neonatal Mortality for Pooled Models, Using Area-Level Measures of Income eTable 7. Estimated Bottom-Top Ratios for Perinatal Mortality, Stillbirths, and Neonatal Mortality for Stratified Models, Using Area-Level Measures of Income eTable 8. Estimated Bottom-Top Ratios for Perinatal Mortality, Stillbirths, and Neonatal Mortality for Pooled Models, Including Only Mothers of Dutch Ethnicity eTable 9. Estimated Bottom-Top Ratios for Perinatal Mortality, Stillbirths, and Neonatal Mortality for Stratified Models, Including Only Mothers of Dutch Ethnicity eTable 10. Estimated Bottom-Top Ratios for Neonatal Mortality, Including Gestational Age as a Mediator [file jamanetwopen-e2132124-s001.pdf]

Supplementary Online Content

Vidiella-Martin J, Been JV, Van Doorslaer E, García-Gómez P, Van Ourti T. Association between income and perinatal mortality in the Netherlands across gestational age. *JAMA Netw Open*. 2021;4(11):e2132124. doi:10.1001/jamanetworkopen.2021.32124

eFigure 1. Cohort Composition

eFigure 2. Distribution of Gestational Age and Birth Weight Centile by Household Income Quintile

eFigure 3. Average Gestational Age and Birth Weight Centile per Household Income Rank and Predicted Outcomes of the Fitted Model

eTable 1. Descriptive Statistics for Unlinked Observations

eTable 2. Estimated Bottom-Top Quintile Ratios for Perinatal Mortality, Stillbirths, and Neonatal Mortality for the Pooled Models

eTable 3. Estimated Bottom-Top Quintile Ratios for Perinatal Mortality, Stillbirths, and Neonatal Mortality From the Stratified Analysis

eTable 4. Beta Coefficients From Logistic Regressions on Perinatal Mortality, Stillbirth, and Neonatal Mortality for Pooled Models

eTable 5. Beta Coefficients From Logistic Regressions on Perinatal Mortality, Stillbirth, and Neonatal Mortality for Stratified Models

eTable 6. Estimated Bottom-Top Ratios for Perinatal Mortality, Stillbirths, and Neonatal Mortality for Pooled Models, Using Area-Level Measures of Income

eTable 7. Estimated Bottom-Top Ratios for Perinatal Mortality, Stillbirths, and Neonatal Mortality for Stratified Models, Using Area-Level Measures of Income

eTable 8. Estimated Bottom-Top Ratios for Perinatal Mortality, Stillbirths, and Neonatal Mortality for Pooled Models, Including Only Mothers of Dutch Ethnicity

eTable 9. Estimated Bottom-Top Ratios for Perinatal Mortality, Stillbirths, and Neonatal Mortality for Stratified Models, Including Only Mothers of Dutch Ethnicity

eTable 10. Estimated Bottom-Top Ratios for Neonatal Mortality, Including Gestational Age as a Mediator

This supplementary material has been provided by the authors to give readers additional information about their work.

© 2021 Vidiella-Martin J et al. *JAMA Network Open.*

eFigure 1. Cohort Composition

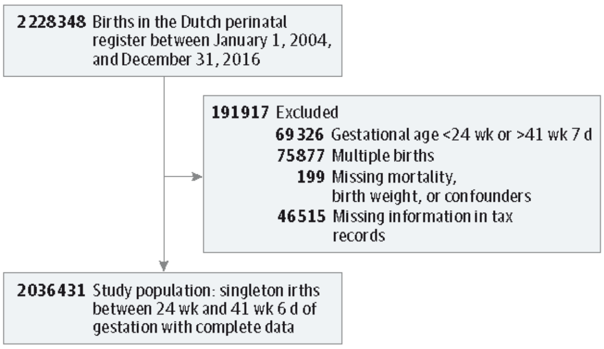

© 2021 Vidiella-Martin J et al. *JAMA Network Open.*

Adjusted gradients for gestational age and birth weight centile

eFigure 2: Distribution of gestational age and birth weight centile by household income quintile

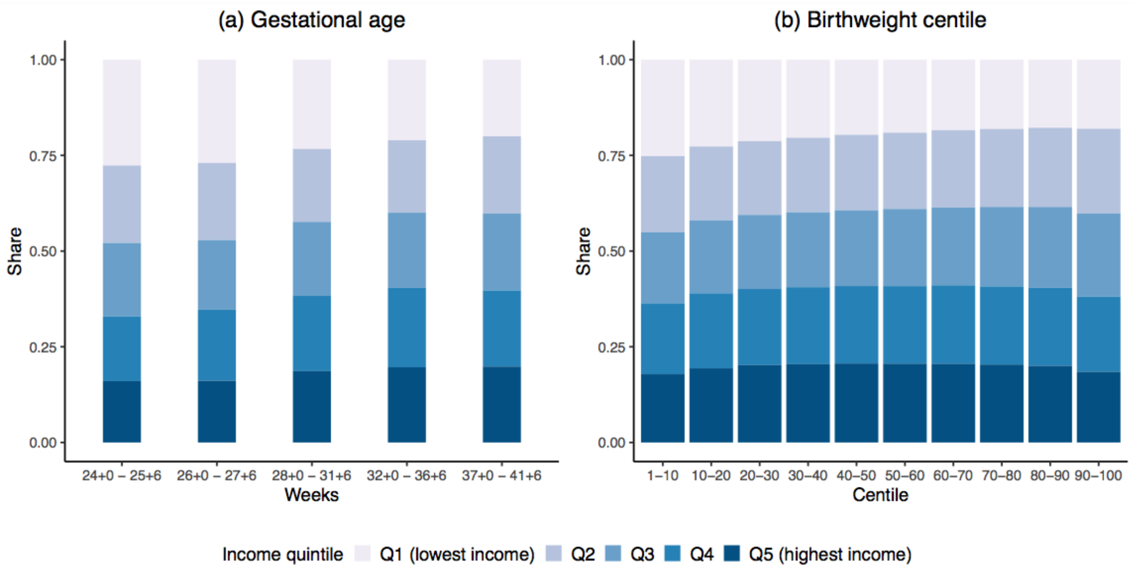

Vertical bars in subfigure (a) show the relative composition of each gestational age category. The lightest colors represent the lowest income quintile, while the darkest ones depict the highest ones. Vertical bars in subfigure (b) capture the relative composition of birth weightdeciles.

eFigure 3: Average gestational age and birth weight centile per household income rank and predicted outcomes of the fitted models

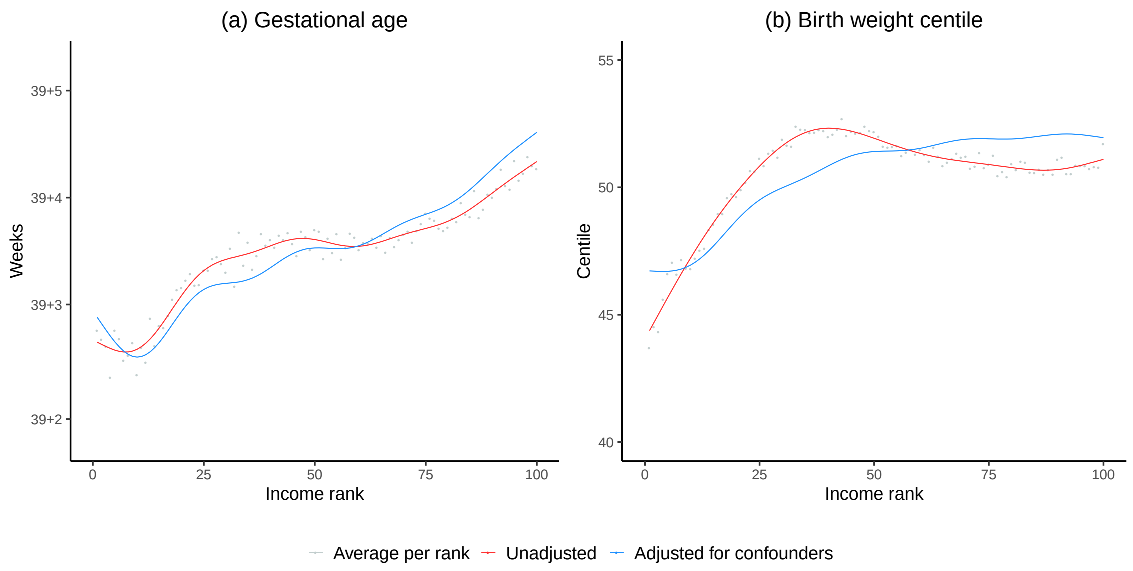

Each dot depicts the average mortality for each household income rank. Predicted outcomes are displayed in solid lines for the model before adjustment (in red) and after adjusted for confounders (in blue).

© 2021 Vidiella-Martin J et al. *JAMA Network Open.*

Information on unlinked births

eTable 1: Descriptive statistics for unlinked births

	Unlinked births (N=46,515)
Maternal age at birth [a]	29.1 (5.8)
<24 [b]	9,168 (19.7)
24-34 [b]	28,294 (60.8)
35-39 [b]	7,325 (15.7)
>39 [b]	1,728 (3.7)
Primiparous [b]	25,152 (54.1)
Ethnicity	
Dutch [b]	13,111 (28.2)
Moroccan [b]	2,163 (4.7)
Turkish [b]	2,162 (4.6)
Surinamese [b]	1,244 (2.7)
Antillean [b]	980 (2.1)
Other nonwestern [b]	13,572 (29.2)
Western [b]	13,283 (28.6)
Female [b]	22,648 (48.7)
Gestational age (weeks) [a]	39.5 (1.9)
24+0 - 25+6 [b]	88 (0.2)
26+0 - 27+6 [b]	92 (0.2)
28+0 - 31+6 [b]	317 (0.7)
32+0 - 36+6 [b]	2,380 (5.1)
37+0 - 41+6 [b]	43,638 (93.8)
Birthweight centile [a]	44.2 (29.4)
BWC < 3 [b,c]	1,911 (4.1)
BWC < 10 [b,c]	6,802 (14.6)
BWC > 90 [b,c]	3,597 (7.7)
Perinatal mortality [d]	367 (7.9)
Stillbirth [d]	275 (5.9)
Neonatal death [d]	92 (2.0)

[a] Mean (SD)
[b] Total number (%)
[c] BWC, birth weight centile
[d] Total number (‰)

© 2021 Vidiella-Martin J et al. *JAMA Network Open.*

Sensitivity analyses

eTable 2: Estimated Q1/Q5 for perinatal mortality, stillbirths, and neonatal mortality for the pooled models

	Unadjusted	Adjusted	Adjusted + BWC[a]
Perinatal mortality	1.72 (1.62 to 1.83)	1.52 (1.44 to 1.61)	1.20 (1.15 to 1.25)
Stillbirth	1.83 (1.70 to 1.96)	1.54 (1.45 to 1.65)	1.19 (1.15 to 1.25)
Neonatal mortality	1.47 (1.32 to 1.63)	1.34 (1.22 to 1.50)	1.16 (1.07 to 1.27)

[a] BWC, birth weight centile
Q1/Q5 ratios divide predicted perinatal mortality, stillbirth, and neonatal mortality for the lowest household income quintile (Q1) by the predicted one for the highest household income rank (Q5). 95% confidence intervals in brackets. Each column corresponds to model: (i) unadjusted; (ii) adjusted for confounding factors (maternal age at delivery, maternal ethnicity, parity, sex, and year of birth); and (iii) after additionally including birth weight centile. Values larger (smaller) than 1 imply that the lowest income quintile households face higher (lower) mortality rates than the highest income quintile ones. A value of 1 indicates no difference between outcome variables for the lowest and highest income quintiles.

eTable 3: Estimated Q1/Q5 ratios for perinatal mortality, stillbirths, and neonatal mortality from the stratified analysis

(a) Perinatal mortality			
	Unadjusted	Adjusted	Adjusted + BWC[a]
24+0 - 25+6	0.93 (0.86 to 1.00)	0.97 (0.90 to 1.07)	0.89 (0.80 to 1.00)
26+0 - 27+6	1.28 (1.13 to 1.47)	1.37 (1.16 to 1.65)	1.12 (0.99 to 1.27)
28+0 - 31+6	1.52 (1.35 to 1.73)	1.39 (1.20 to 1.64)	1.19 (1.07 to 1.34)
32+0 - 36+6	2.00 (1.79 to 2.23)	1.55 (1.34 to 1.79)	1.18 (1.08 to 1.32)
37+0 - 41+6	1.41 (1.29 to 1.54)	1.35 (1.26 to 1.48)	1.17 (1.11 to 1.25)
(b) Stillbirth			
	Unadjusted	Adjusted	Adjusted + BWC[a]
24+0 - 25+6	1.05 (0.93 to 1.19)	1.13 (0.98 to 1.32)	0.99 (0.86 to 1.15)
26+0 - 27+6	1.52 (1.29 to 1.82)	1.67 (1.36 to 2.11)	1.18 (1.05 to 1.33)
28+0 - 31+6	1.64 (1.42 to 1.90)	1.49 (1.26 to 1.80)	1.20 (1.09 to 1.37)
32+0 - 36+6	1.99 (1.75 to 2.25)	1.58 (1.36 to 1.87)	1.19 (1.08 to 1.33)
37+0 - 41+6	1.42 (1.28 to 1.57)	1.34 (1.23 to 1.49)	1.17 (1.09 to 1.26)
(c) Neonatal mortality			
	Unadjusted	Adjusted	Adjusted + BWC[a]
24+0 - 25+6	0.76 (0.64 to 0.90)	0.82 (0.66 to 1.00)	0.80 (0.64 to 0.97)
26+0 - 27+6	0.92 (0.68 to 1.27)	0.73 (0.38 to 1.28)	0.73 (0.42 to 1.17)
28+0 - 31+6	1.25 (0.97 to 1.65)	1.03 (0.65 to 1.61)	1.02 (0.71 to 1.49)
32+0 - 36+6	2.07 (1.66 to 2.63)	1.44 (1.00 to 2.05)	1.15 (0.86 to 1.56)
37+0 - 41+6	1.37 (1.17 to 1.63)	1.38 (1.19 to 1.66)	1.17 (1.04 to 1.34)

[a] BWC, birth weight centile
Q1/Q5 ratios divide predicted perinatal mortality, stillbirth, and neonatal mortality for the lowest household income quintile (Q1) by the predicted one for the highest household income rank (Q5). 95% confidence intervals in brackets. Each column corresponds to model: (i) unadjusted; (ii) adjusted for confounding factors (maternal age at delivery, maternal ethnicity, parity, sex, and year of birth); and (iii) after additionally including birth weight centile. Values larger (smaller) than 1 imply that the lowest income quintile households face higher (lower) mortality rates than the highest income quintile ones. A value of 1 indicates no difference between outcome variables for the lowest and highest income quintiles.

© 2021 Vidiella-Martin J et al. *JAMA Network Open.*

© 2021 Vidiella-Martin J et al. *JAMA Network Open.*

eTable 4: Odds ratios from logistic regressions on perinatal mortality, stillbirth, and neonatal mortality

	Unadjusted	Adjusted	Adjusted + BWC[a]
Perinatal mortality	0.9936 (0.9929 to 0.9942)	0.9922 (0.9915 to 0.993)	0.9939 (0.9932 to 0.9947)
Stillbirth	0.9929 (0.9921 to 0.9937)	0.9912 (0.9903 to 0.9921)	0.9933 (0.9924 to 0.9941)
Neonatal mortality	0.9953 (0.9941 to 0.9966)	0.9949 (0.9935 to 0.9963)	0.9957 (0.9944 to 0.9972)

[a] BWC, birth weight centile

Each column corresponds to a different model: (i) unadjusted; (ii) adjusted for confounding factors (maternal age at delivery, maternal ethnicity, parity, sex, and year of birth); and (iii) after including birth weight centile.

eTable 5: Odds ratios from logistic regressions on perinatal mortality, stillbirth, and neonatal mortality by gestational age strata

(a) Perinatal mortality			
	Unadjusted	Adjusted	Adjusted + BWC[a]
24+0 - 25+6	1.0027 (0.9999 to 1.0054)	1.0009 (0.9977 to 1.0042)	1.0030 (0.9995 to 1.0065)
26+0 - 27+6	0.9956 (0.9931 to 0.9981)	0.9951 (0.9923 to 0.9980)	0.9970 (0.9940 to 1.0000)
28+0 - 31+6	0.9941 (0.9923 to 0.9959)	0.9956 (0.9935 to 0.9976)	0.9963 (0.9942 to 0.9984)
32+0 - 36+6	0.9910 (0.9896 to 0.9925)	0.9938 (0.9921 to 0.9954)	0.9953 (0.9936 to 0.9969)
37+0 - 41+6	0.9958 (0.9947 to 0.9968)	0.9941 (0.9929 to 0.9953)	0.9952 (0.9940 to 0.9964)
(b) Stillbirth			
	Unadjusted	Adjusted	Adjusted + BWC[a]
24+0 - 25+6	0.9989 (0.9962 to 1.0016)	0.9976 (0.9946 to 1.0007)	0.9998 (0.9963 to 1.0034)
26+0 - 27+6	0.9936 (0.9908 to 0.9963)	0.9924 (0.9892 to 0.9956)	0.9944 (0.9911 to 0.9978)
28+0 - 31+6	0.9933 (0.9912 to 0.9953)	0.9942 (0.9919 to 0.9966)	0.9952 (0.9929 to 0.9976)
32+0 - 36+6	0.9912 (0.9895 to 0.9928)	0.9930 (0.9912 to 0.9949)	0.9949 (0.9930 to 0.9968)
37+0 - 41+6	0.9956 (0.9943 to 0.9969)	0.9937 (0.9923 to 0.9952)	0.9949 (0.9934 to 0.9963)
(c) Neonatal mortality			
	Unadjusted	Adjusted	Adjusted + BWC[a]
24+0 - 25+6	1.0056 (1.0020 to 1.0092)	1.0038 (0.9996 to 1.0081)	1.0042 (1.0000 to 1.0085)
26+0 - 27+6	1.0011 (0.9968 to 1.0054)	1.0025 (0.9975 to 1.0075)	1.0031 (0.9981 to 1.0081)
28+0 - 31+6	0.9971 (0.9936 to 1.0007)	1.0000 (0.9959 to 1.0041)	0.9997 (0.9956 to 1.0038)
32+0 - 36+6	0.9909 (0.9881 to 0.9936)	0.9959 (0.9927 to 0.9990)	0.9965 (0.9934 to 0.9996)

© 2021 Vidiella-Martin J et al. *JAMA Network Open.*

37+0 - 41+6	0.9960 (0.9941 to 0.9980)	0.9950 (0.9928 to 0.9972)	0.9961 (0.9939 to 0.9983)

[a] BWC, birth weight centile

Each column corresponds to a different model: (i) unadjusted; (ii) adjusted for confounding factors (maternal age at delivery, maternal ethnicity, parity, sex, and year of birth); and (iii) after including birth weight centile.

© 2021 Vidiella-Martin J et al. *JAMA Network Open.*

eTable 6: Estimated bottom-to-top ratios for perinatal mortality, stillbirths, and neonatal mortality, using area-level measures of income rank

	Unadjusted	Adjusted	Adjusted + BWC[a]
Perinatal mortality	1.63 (1.48 to 1.79)	1.39 (1.32 to 1.48)	1.15 (1.11 to 1.20)
Stillbirth	1.68 (1.50 to 1.88)	1.42 (1.33 to 1.51)	1.14 (1.10 to 1.19)
Neonatal mortality	1.47 (1.26 to 1.72)	1.32 (1.16 to 1.50)	1.15 (1.04 to 1.29)

[a] BWC, birth weight centile

Bottom-to-top ratios divide predicted perinatal mortality, stillbirth, and neonatal mortality for the lowest household income rank (1) by the predicted one for the highest household income rank (100). 95% confidence intervals in parentheses. Each column corresponds to model: (i) unadjusted; (ii) adjusted for confounding factors (maternal age at delivery, maternal ethnicity, parity, sex, and year of birth); and (iii) after additionally including birth weight centile. Values larger (smaller) than 1 imply that the lowest income households face higher (lower) mortality rates than the highest income ones. A value of 1 indicates no difference between outcome variables for the lowest and highest household income ranks.

eTable 7: Estimated bottom-to-top ratios for perinatal mortality, stillbirths, and neonatal mortality by gestational age strata, using area-level measures of income rank

(a) Perinatal mortality			
	Unadjusted	Adjusted	Adjusted + BWC[a]
24+0 - 25+6	0.96 (0.88 to 1.06)	1.01 (0.90 to 1.11)	0.94 (0.83 to 1.07)
26+0 - 27+6	1.43 (1.22 to 1.69)	1.49 (1.22 to 1.84)	1.21 (1.05 to 1.41)
28+0 - 31+6	1.61 (1.38 to 1.89)	1.43 (1.21 to 1.74)	1.24 (1.10 to 1.43)
32+0 - 36+6	2.16 (1.89 to 2.51)	1.55 (1.32 to 1.88)	1.21 (1.08 to 1.38)
37+0 - 41+6	1.27 (1.13 to 1.42)	1.24 (1.13 to 1.36)	1.10 (1.03 to 1.18)
(b) Stillbirth			
	Unadjusted	Adjusted	Adjusted + BWC[a]
24+0 - 25+6	1.09 (0.94 to 1.29)	1.13 (0.95 to 1.34)	1.02 (0.85 to 1.23)
26+0 - 27+6	1.64 (1.34 to 2.06)	1.75 (1.36 to 2.28)	1.24 (1.09 to 1.46)
28+0 - 31+6	1.73 (1.47 to 2.09)	1.51 (1.25 to 1.87)	1.23 (1.10 to 1.44)
32+0 - 36+6	2.08 (1.78 to 2.49)	1.53 (1.29 to 1.86)	1.18 (1.06 to 1.34)
37+0 - 41+6	1.25 (1.08 to 1.43)	1.22 (1.12 to 1.36)	1.10 (1.02 to 1.19)
(c) Neonatal mortality			
	Unadjusted	Adjusted	Adjusted + BWC[a]
24+0 - 25+6	0.81 (0.67 to 1.01)	0.88 (0.69 to 1.10)	0.86 (0.67 to 1.07)
26+0 - 27+6	1.09 (0.75 to 1.60)	0.94 (0.46 to 1.79)	0.92 (0.51 to 1.56)
28+0 - 31+6	1.36 (1.00 to 1.91)	1.14 (0.69 to 1.96)	1.11 (0.72 to 1.78)
32+0 - 36+6	2.43 (1.84 to 3.24)	1.66 (1.12 to 2.91)	1.32 (0.93 to 1.91)
37+0 - 41+6	1.33 (1.06 to 1.65)	1.30 (1.07 to 1.62)	1.11 (0.97 to 1.32)

[a] BWC, birth weight centile

Bottom-to-top ratios divide predicted perinatal mortality, stillbirth, and neonatal mortality for the lowest household income rank (1) by the predicted one for the highest household income rank (100). 95% confidence intervals in parentheses. Each column corresponds to model: (i) unadjusted; (ii) adjusted for confounding factors (maternal age at delivery, maternal ethnicity, parity, sex, and year of birth); and (iii) after additionally including birth weight centile. Values larger (smaller) than 1 imply that the lowest income households face higher (lower) mortality rates than the highest income ones. A value of 1 indicates no difference between outcome variables for the lowest and highest household income ranks.

© 2021 Vidiella-Martin J et al. *JAMA Network Open.*

eTable 8: Estimated bottom-to-top ratios for perinatal mortality, stillbirths, and neonatal mortality, including only Dutch mothers

	Unadjusted	Adjusted	Adjusted + BWC[a]
Perinatal mortality	1.77 (1.55 to 2.02)	1.63 (1.50 to 1.79)	1.17 (1.10 to 1.24)
Stillbirth	1.99 (1.66 to 2.38)	1.70 (1.53 to 1.91)	1.17 (1.10 to 1.26)
Neonatal mortality	1.34 (1.14 to 1.59)	1.44 (1.23 to 1.72)	1.14 (1.01 to 1.30)

[a] BWC, birth weight centile

Bottom-to-top ratios divide predicted perinatal mortality, stillbirth, and neonatal mortality for the lowest household income rank (1) by the predicted one for the highest household income rank (100). 95% confidence intervals in parentheses. Each column corresponds to model: (i) unadjusted; (ii) adjusted for confounding factors (maternal age at delivery, maternal ethnicity, parity, sex, and year of birth); and (iii) after additionally including birth weight centile. Values larger (smaller) than 1 imply that the lowest income households face higher (lower) mortality rates than the highest income ones. A value of 1 indicates no difference between outcome variables for the lowest and highest household income ranks.

eTable 9: Estimated bottom-to-top ratios for perinatal mortality, stillbirths, and neonatal mortality gestational age strata, including only Dutch mothers

(a) Perinatal mortality			
	Unadjusted	Adjusted	Adjusted + BWC[a]
24+0 - 25+6	0.95 (0.84 to 1.07)	0.93 (0.81 to 1.07)	0.82 (0.70 to 0.97)
26+0 - 27+6	1.37 (1.11 to 1.70)	1.24 (0.96 to 1.61)	1.03 (0.83 to 1.26)
28+0 - 31+6	1.51 (1.26 to 1.88)	1.46 (1.08 to 1.94)	1.17 (0.99 to 1.41)
32+0 - 36+6	2.11 (1.77 to 2.57)	1.74 (1.39 to 2.24)	1.21 (1.02 to 1.45)
37+0 - 41+6	1.28 (1.11 to 1.48)	1.42 (1.26 to 1.61)	1.14 (1.05 to 1.26)
(b) Stillbirth			
	Unadjusted	Adjusted	Adjusted + BWC[a]
24+0 - 25+6	1.17 (0.98 to 1.42)	1.13 (0.93 to 1.40)	0.93 (0.75 to 1.18)
26+0 - 27+6	1.64 (1.20 to 2.24)	1.54 (1.09 to 2.21)	1.09 (0.93 to 1.32)
28+0 - 31+6	1.65 (1.33 to 2.12)	1.69 (1.21 to 2.56)	1.23 (1.04 to 1.49)
32+0 - 36+6	2.10 (1.69 to 2.66)	1.77 (1.39 to 2.39)	1.22 (1.06 to 1.46)
37+0 - 41+6	1.27 (1.07 to 1.52)	1.42 (1.24 to 1.64)	1.17 (1.06 to 1.30)
(c) Neonatal mortality			
	Unadjusted	Adjusted	Adjusted + BWC[a]
24+0 - 25+6	0.71 (0.55 to 0.93)	0.75 (0.56 to 1.00)	0.74 (0.57 to 0.98)
26+0 - 27+6	1.04 (0.66 to 1.72)	0.74 (0.30 to 1.60)	0.70 (0.28 to 1.45)
28+0 - 31+6	1.23 (0.83 to 1.91)	0.99 (0.45 to 2.16)	0.96 (0.53 to 1.80)
32+0 - 36+6	2.17 (1.54 to 3.17)	1.55 (0.83 to 3.20)	1.10 (0.67 to 1.86)
37+0 - 41+6	1.29 (1.02 to 1.68)	1.42 (1.11 to 1.89)	1.10 (0.93 to 1.38)

[a] BWC, birth weight centile

Bottom-to-top ratios divide predicted perinatal mortality, stillbirth, and neonatal mortality for the lowest household income rank (1) by the predicted one for the highest household income rank (100). 95% confidence intervals in parentheses. Each column corresponds to model: (i) unadjusted; (ii) adjusted for confounding factors (maternal age at delivery, maternal ethnicity, parity, sex, and year of birth); and (iii) after additionally including birth weight centile. Values larger (smaller) than 1 imply that the lowest income households face higher (lower) mortality rates than the highest income ones. A value of 1 indicates no difference between outcome variables for the lowest and highest household income ranks.

© 2021 Vidiella-Martin J et al. *JAMA Network Open.*

© 2021 Vidiella-Martin J et al. *JAMA Network Open.*

eTable 10: Estimated bottom-to-top ratios for neonatal mortality, including gestational age as a mediator

	Adjusted + GA[a]	Adjusted + GA + BWC[b]
Neonatal mortality	1.07 (1.02 to 1.14)	1.03 (0.98 to 1.08)

[a] GA, gestational age

[b] BWC, birth weight centile

Bottom-to-top ratios divide predicted perinatal mortality, stillbirth, and neonatal mortality for the lowest household income rank (1) by the predicted one for the highest household income rank (100). 95% confidence intervals in parentheses. Each column corresponds to model: (i) adjusted for confounding factors (maternal age at delivery, maternal ethnicity, parity, sex, and year of birth) and including gestational age; and (iii) after additionally including birth weight centile. Values larger (smaller) than 1 imply that the lowest income households face higher (lower) mortality rates than the highest income ones. A value of 1 indicates no difference between outcome variables for the lowest and highest household income ranks.

© 2021 Vidiella-Martin J et al. *JAMA Network Open.*
